# The Potential and Action Mechanism of Polyphenols in the Treatment of Liver Diseases

**DOI:** 10.1155/2018/8394818

**Published:** 2018-02-04

**Authors:** Sha Li, Hor Yue Tan, Ning Wang, Fan Cheung, Ming Hong, Yibin Feng

**Affiliations:** ^1^School of Chinese Medicine, The University of Hong Kong, Pok Fu Lam, Hong Kong; ^2^Shenzhen Institute of Research and Innovation, Pok Fu Lam, The University of Hong Kong, Hong Kong; ^3^Institute of Clinical Pharmacology, Guangzhou University of Chinese Medicine, Guangzhou, China

## Abstract

Liver disease, involving a wide range of liver pathologies from fatty liver, hepatitis, and fibrosis to cirrhosis and hepatocellular carcinoma, is a serious health problem worldwide. In recent years, many natural foods and herbs with abundant phytochemicals have been proposed as health supplementation for patients with hepatic disorders. As an important category of phytochemicals, natural polyphenols have attracted increasing attention as potential agents for the prevention and treatment of liver diseases. The striking capacities in remitting oxidative stress, lipid metabolism, insulin resistance, and inflammation put polyphenols in the spotlight for the therapies of liver diseases. It has been reported that many polyphenols from a wide range of foods and herbs exert therapeutic effects on liver injuries via complicated mechanisms. Therefore, it is necessary to have a systematical review to sort out current researches to help better understand the potentials of polyphenols in liver diseases. In this review, we aim to summarize and update the existing evidence of natural polyphenols in the treatment of various liver diseases by *in vitro*, *in vivo*, and clinical studies, while special attention is paid to the action mechanisms.

## 1. Introduction

Liver diseases, containing a wide range of hepatic pathologies from steatosis, hepatitis, and cirrhosis to hepatocellular carcinoma (HCC), are leading causes of morbidity and mortality worldwide and have caused huge socioeconomic burdens [1]. The main etiologies of liver diseases are alcohol abuse, hepatitis virus infections, and metabolic syndrome [1]. The most important pathological processes of liver diseases are oxidative stress, lipid peroxidation, inflammation, and immune response disruption [2]. In response to hepatic injury, a cascade of molecular and cellular reactions would be generated with the aims of restraining damage, repairing damaged cells and tissues, defensing against further infection, and regeneration. Inflammation in the hepatic injury, the primary response, may initiate myofibroblast differentiation and activation that produce fibrous tissue and induce parenchymal cell proliferation, resulting in fibrosis and ultimately cirrhosis, the platform on which HCC and deadly hepatic failure develop [3]. As a matter of fact, most of the unresolved challenges in hepatology could be attributed to an imbalance of inflammatory processes [4, 5]. On one hand, chronic hepatic inflammation promotes the progression of liver diseases, for example, from fatty liver to steatohepatitis. On the other hand, insufficient antimicrobial responses, inadequate tumor clearance, and/or suppression of antitumor immunity in the liver of patients with end-stage cirrhosis would lead to life-threatening bacterial infections and HCC development [4]. More importantly, oxidative stress, lipid peroxidation, and immune disorder have a close relationship with hepatic inflammation, which leads to an extremely complex network involved in the pathogenesis of liver diseases. The multiple pathways involved in the pathogenesis have provided extensive therapeutic targets for potential treatments [6].

Natural polyphenols are secondary metabolites of plants, which become noticeable as potential agents for prevention and treatment of several diseases, such as cancer, cardiovascular diseases, diabetes mellitus, aging, and neurodegenerative diseases [7]. They usually have subtle effects on multiple targets that eventually result in significant health benefits. Strikingly, polyphenols have been found to possess a variety of pharmacological effects on oxidative stress, lipid metabolism, insulin resistance, and inflammation, which are the most important pathological processes in the etiology of liver diseases [7, 8]. This puts polyphenols under spotlight for the therapy of liver diseases. In this review, we summarize the distribution of polyphenols in natural products to offer guidance for drug/health product development and dietary supplementation and focus on updating the existing animal and clinical trial results for the use of polyphenols in the treatment of liver diseases in different stages.

## 2. Polyphenols in Natural Products

**Figure 1:**
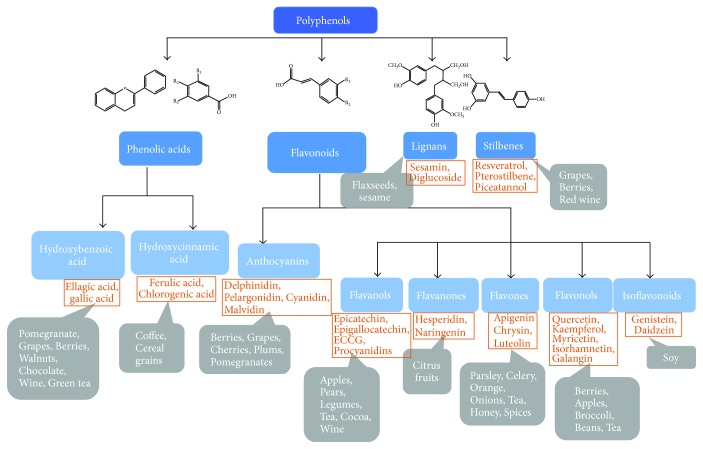
The classification and major dietary source of natural polyphenols. The four dark blue rectangles represent four major categories of polyphenols, while light blue rectangles are subcategories within the major classifications. The orange rectangles are representative polyphenols for each subcategory, and gray rectangles are major dietary sources for the corresponding representative polyphenols.

Natural polyphenols is a large group of plant secondary metabolites ranging from small molecules to highly polymerized compounds, having at least one aromatic ring with one or more hydroxyl functional groups attached [9]. Based on chemical structures, natural polyphenols can be chemically divided into several classes, including flavonoids, phenolic acids, lignans, stilbenes, and other polyphenols [9, 10]. Among them, flavonoids and phenolic acids account for about 60% and 30% of all natural polyphenols, respectively. Natural polyphenols are ubiquitously present in nature and particularly have been found in high quantities in many foods and plants, such as vegetables, fruits, cereals, spices, mushrooms, tea, microalgae, medical plants, wild fruits, and flowers [7]. The representative members and major dietary sources of each class are briefly summarized in Figure 1. A variety of factors, including but not limited to environmental condition, genotype, cultivar, harvest time, storage, and processing, could affect the levels of polyphenols in foods and plants, while species is still considered to be the primary factor resulting in different quantities in different products. By comparing the contents of polyphenols in a great deal of natural product through our studies and literature retrieval, several representative species with relatively high quantities of polyphenols in different kinds of natural products are listed in Table 1. In general, spices, medicinal plants, and fruit peels contain comparatively abundant polyphenols, which deserve special attention for further extraction, separation, and identification of phenolic compounds.

## 3. The Potential and Mechanism of Action of Polyphenols in the Treatment of Liver Diseases

### 3.1. Liver Injury Induced by Toxins and Drugs

Liver is a central organ responsible for the metabolism of drugs and toxic chemicals, and thus it is the primary target organ for various exogenous toxins, such as alcohol, organic solvents, heavy metals, and drugs [1]. As the main pathogenic mechanisms responsible for those toxic damages are oxidative stress, inflammation, dysfunction of cytochrome P450, and mitochondrial dysfunction [22], the application of flavonoids in attenuating liver injury induced by these toxins has been extensively studied. A wide spectrum of flavonoids showed promising therapeutic effects on liver injury induced by various toxins using animal models. The underlying mechanisms mainly involve enhancing antioxidative defense enzymes via mediating nuclear factor erythroid 2-related factor 2 (Nrf2)/cytochrome P450 2E1 (CYP2E1) expression, alleviating inflammation by inactivation of mitogen-activated protein kinase (MAPK)/nuclear factor kappaB (NF-*κ*B) signaling pathways and reducing apoptosis through regulating B-cell lymphoma 2 (Bcl-2)/protein kinase B (AKT)/caspase expression.

Carbon tetrachloride- (CCl_4_-) induced hepatotoxicity has been widely investigated in hepatology. Covalent binding of the CCl_3_^∗^ radical to cell components inhibits lipoprotein secretion and thus initiates steatosis, whereas reaction with oxygen to form CCl_3_-OO^∗^, introduces lipid peroxidation which in consequence results in apoptosis and cell death. Quercetin, a natural flavonoid with many beneficial effects, significantly protected liver from CCl_4_-induced injury via antioxidative stress and anti-inflammation. The underlying mechanism was ascribed to the inhibition of Toll-like receptor 2 (TLR2) and Toll-like receptor 4 (TLR4) activations and MAPK phosphorylation, leading to inactivation of NF-*κ*B and in turn reduced hepatic inflammatory cytokines [23]. Puerarin, a natural flavonoid that has been reported to have various medicinal properties, also remarkably attenuated CCl_4_-induced hepatotoxicity by reducing ROS production, renewing the antioxidant enzyme system and regulating expression of hepatic lipid biosynthesis and metabolism genes. It could restore total antioxidant capacity and GSH levels and significantly inhibit hyperlipidemia via regulating the expression of phosphorylated Jun N-terminal kinases (JNK), phosphorylated c-Jun protein, and cholesterol 7a-hydroxylase (CYP7A1) in the liver of mice receiving CCl_4_ [24]. Additionally, a marine polyphenol, dieckol, was found to be against CCl_4_-induced liver damage in mice via mediating apoptosis-regulating genes including downregulation of Bax and upregulation of Bcl-xl protein expressions [25]. In another study, isorhamnetin-3-O-galactoside, a flavonoid glycoside isolated from *Artemisia capillaris* Thunberg, showed protection on CCl_4_-induced hepatic injury through decreasing the level of phosphorylated c-JNK, extracellular signal-regulated kinase (ERK), and p38 MAPK. It diminished the increases of NF-*κ*B and c-Jun nuclear translocation whereas enhanced the nuclear level of Nrf2, indicating its role in enhancing antioxidative defense system and reducing inflammation [26]. The flavonoid fraction from *Rosa laevigata* Michx fruit acted against CCl_4_-induced acute liver injury in mice through downregulating the expression of CYP2E1, inducible nitric oxide synthases (iNOS), NF-*κ*B, Bal, and Caspase-3, which was related to signaling pathways of oxidative stress, inflammation, and apoptosis [27].

Drug-induced liver injury is an important clinical issue. More than 900 drugs affect the liver directly or through mediating an immune response. Acetaminophen (AAP) is a classic example of a known intrinsic hepatotoxin at supertherapeutic dose. Baicalin, a well-known flavonoid of *Scutellariae radix*, can effectively relieve AAP-induced liver injury mainly through downregulating the ERK signaling pathway and its downstream effectors of inflammatory responses [28]. Polyphenol-enriched fraction from the leaves of *Microcos paniculata* L. showed hepatoprotective effect against AAP-induced liver damage via dual regulation of reactive oxygen species (ROS)/MAPKs/apoptosis axis and Nrf2-mediated antioxidant response [29]. A polyphenol extract of *Hibiscus sabdariffa* L. could ameliorate AAP-induced liver steatosis accompanied by a reduced hepatic expression of apoptosis-inducing factor (AIF), Bax, Bid, and p-JNK, suggesting it may exert hepatoprotective effect through attenuating the mitochondrial dysfunction [30].

The protective effects of several polyphenols on other toxins such as lipopolysaccharide (LPS) and thioacetamide (TAA) have also been extensively demonstrated, majorly via antioxidative stress and anti-inflammation. Here, we selected several studies that showed the effects of compounds with well-identified action mechanisms to discuss. Nobiletin, an O-methylated flavone, which is found in rich in the peel of citrus fruits, was able to protect the liver from LPS/D-galactosamine-induced injury through activating the Nrf2 antioxidant pathway and subsequent inhibiting NF-*κ*B-mediated cytokine production [31]. Curcumin, a natural plant phenolic food additive, was found to significantly attenuate LPS-caused liver failure. It decreased serum ALT, AST, and ALP levels, improved antioxidant enzyme levels, and inhibited activation of the mitogen-activated protein kinases/c-Jun NH2-terminal kinase (P38/JNK) cascade in the livers of rats with LPS administration. Furthermore, it reduced serum cytokines such as IL-6, IL-1*β*, and tumor necrosis factor-*α* (TNF-*α*) and improved liver apoptosis via suppression of phosphatidylinositol 3-kinase/protein kinase B (PI3K/AKT) signaling pathway and inhibition of cyclic AMP-responsive element-binding protein (CREB)/caspase expression. In addition, it regulated oxidative stress-associated signaling pathway in LPS-treated mice, as indicated by downregulated CYP2E1/Nrf2/ROS protein expression. Thus, curcumin may serve as a promising candidate to inhibit inflammation and apoptosis signaling for the treatment of endotoxemia-induced liver failure [32]. Silymarin, a mixture of flavonolignans extracted from *Silybum marianum* Gaertneri showed the ability to diminish hepatic lesions and inflammation caused by bisphenol A in mice [33]. Resveratrol, a naturally occurring polyphenol that possesses a variety of pharmacological activities, showed significant hepatoprotective effects on TAA-induced liver injury. It inhibited inflammation and oxidative stress by downregulating NF-*κ*B and CYP2E1 expression and enhanced apoptosis of necrotic hepatocytes through enhancing the activity of caspase-3 [34].

### 3.2. Alcoholic Liver Disease

Alcoholic liver disease (ALD) is one of the most important causes of liver-related death. Although the understanding about the progression and pathogenesis of ALD has been advanced, there are no universally accepted therapies to treat this disease in human at present [1]. The direct consequences of ethanol metabolism are related to ROS production, mitochondrial injury, and hepatic steatosis, which are the common features of acute and chronic alcohol exposures [35]. Alcoholic fatty liver is a reversible condition, but it can potentiate the development of alcoholic hepatitis and cirrhosis by promoting free radical generation. A great deal of polyphenols has been found to be beneficial for alcoholic liver injury associating with hepatic lipid metabolism regulation and antioxidative stress. Total flavonoids from *Litsea coreana* showed therapeutic effects on alcoholic fatty liver via suppression of hepatic adipose differentiation-related protein (ADRP) [36]. The supplementation of a novel flavonoid, fisetin, in the diet at 10 mg/kg/day also showed remarkable beneficial effect on alcohol-induced liver injury. Hepatic NADPH oxidase 4 levels along with plasma hydrogen peroxide and hepatic superoxide and 4-hydroxynonenal levels increased by alcohol consumption were reduced by fisetin supplementation. Fisetin attenuated liver steatosis through enhancing plasma adiponectin levels and hepatic protein expressions of p-AMPK, acyl-CoA oxidase 1 (ACOX1), cytochrome P450 4A (CYP4A), and microsomal triglyceride transfer protein (MTTP) [37]. In a binge drinking mouse model, a polymethoxy flavonoid-rich *Citrus aurantium* extract alleviated alcohol-induced liver injury through activating lipid metabolism-related signals and regulating AMPK and Nrf2-related pathway signaling [38]. In mice model with chronic plus binge alcohol feeding, luteolin attenuated the liver injury via downregulation of lipogenic genes including sterol regulatory element-binding protein 1c (SREBP-1c), fatty acid synthase (FASN), acetyl-CoA carboxylase (ACC), and stearoyl-CoA desaturase (SCD1), suggesting its significant effect on alleviating liver steatosis [39]. In another study, oligomeric proanthocyanidins, a set of bioflavonoid complexes having strong free radical scavenging ability, protected liver from alcohol-induced injury and steatosis through decreasing the expressions of lipid synthesis genes and inflammation genes including SREBP-1c, SREBP2, interleukin 1 beta (IL-1*β*), IL-6, and TNF-*α*, indicating that AMPK activation might be involved in the underlying mechanism [40].

During the metabolic processes of alcohol in the liver via dehydrogenase system and microsomal ethanol oxidizing system (MEOS), NADH or NADP^+^ are generated in bulk, with the consequence of increased ROS, eventually resulting in cellular and tissue injury [1]. With better understanding of the role of oxidative stress in the initiation and advancement of ALD, therapies targeting on enhancing antioxidant defense have been considered promising. In an *in vitro* study, it was found that *Ecklonia cava* polyphenol served as a promising candidate for inhibiting alcohol-induced hepatic damage via regulating alcohol metabolic enzymes including CYP2E1 and ADH in a cyclic AMP-dependent manner [41]. Two ellagitannins, geraniin and amariin, which belong to a type of polyphenol formed mainly from the oxidative linkage of galloyl groups in 1,2,3,4,6-pentagalloyl glucose, were isolated from *Phyllanthus amarus*. It was found that both of them could protect mouse liver from alcoholic cytotoxicity through restoring antioxidant enzymes, inhibiting oxidation of lipid and protein, ceasing formation of 8-hydroxy-2-deoxyguanosine, and modulating Bcl-2-associated X (Bax)/Bcl2 ratio against apoptosis [42].

Regarding action mechanisms of polyphenols in ALD, in addition to regulating hepatic steatosis and antioxidative stress, several other mechanisms have also been proposed. For example, it was demonstrated that polyphenols could suppress the expression of genes related to cell stress and upregulate genes involved in bile acid synthesis, unsaturated fatty acid elongation, and tetrahydrofolate synthesis [43]. Liver iron overload has long been considered as pathogenic factors of ALD. Iron is involved in the Fenton pathway, and it accumulates during chronic hepatic inflammation and catalyzes hydroxyl radical-mediated oxidative injury [44]. The deposition of iron in the liver may increase the risk of death in patients with ALD [44]. Therefore, removal of iron represents an important therapeutic strategy for ALD treatment. In a study, epigallocatechin-3-gallate (EGCG) has been demonstrated to ameliorate alcoholic liver injuries associated with its iron-chelating property. It affected hepatic iron uptake and inhibited iron absorption in the small intestinal via upregulating hepcidin mRNA levels and transferrin as well as hepatic transferrin receptor protein levels, thus reducing serum and hepatic iron levels [45].

### 3.3. NAFLD

Nonalcoholic fatty liver disease (NAFLD), defined as genetic-environmental-metabolic stress-related disease with a spectrum of liver disorders, affects 10% to 24% of the population worldwide, and the prevalence has even been up to 75% in obese people [46]. Currently, no evidence-based pharmacological therapy is available for NAFLD. A multitude of pathways implicated in the etiology of NAFLD makes the treatment challenging. Ideally, the treatment should address all these pathways [47]. Free fatty acids, oxidative stress, and inflammation that cause insulin resistance, hepatocyte fat accumulation, and cellular injury are the major processes involved in the progression of NAFLD [47]. Reasonably, polyphenols with remarkable ability in metabolism regulation, antioxidant, and anti-inflammation have been considered as promising therapies of NAFLD. The mechanisms underlying beneficial effects of many polyphenols on NAFLD have been extensively studied in recent years. In addition to regulation of classical intracellular signaling transduction, some of them were demonstrated to exert therapeutic effects via emerging mechanisms such as mediating microRNAs and gut microbiota regulation.

#### 3.3.1. Intracellular Signaling Transduction

**Figure 2:**
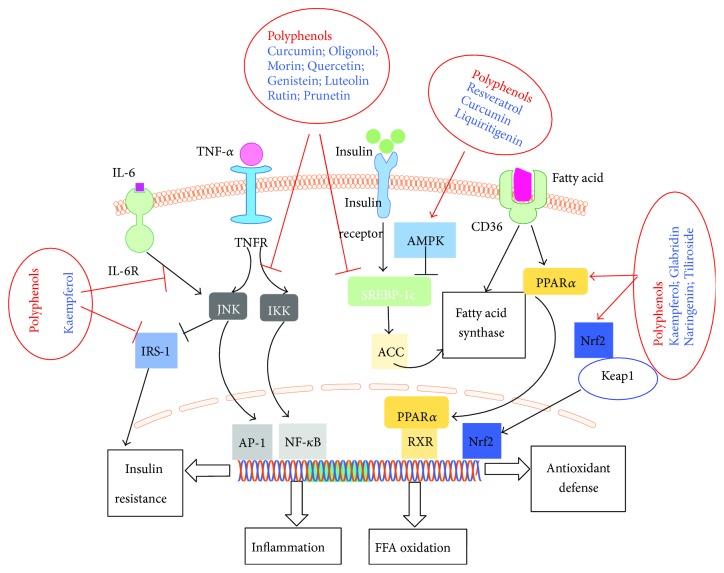
Intracellular signaling transduction mediated by polyphenols for the treatment of NAFLD. Polyphenols may prevent injury in hepatocytes associated with NAFLD through several signaling pathways: (1) suppressing activation of NF-*κ*B pathway to inhibit inflammation; (2) increasing *β*-fatty acid oxidation by upregulating PPAR*α*; (3) inhibiting lipogenesis via downregulation of SREBP-1c by AMPK activation; and (4) enhancing antioxidant defense through Nrf2 pathway.

Signaling pathways are associated with insulin resistance, oxidative stress, and inflammation include NF-*κ*B, AMPK, Janus kinase/signal transducers and activators of transcription (JAK/STAT), peroxisome proliferator-activated receptors (PPARs), SREBP-1c, phosphatidylinositol 3-kinase/protein kinase B (PI3K/Akt), and TLR [5]. Blocking the transmission of above pathways within the liver cells would be effective for the prevention and treatment of NAFLD. Polyphenols may prevent hepatocyte injury associated with NAFLD through several signaling pathways: (1) suppressing activation of NF-*κ*B pathway to inhibit inflammation; (2) increasing *β*-fatty acid oxidation by upregulating PPAR*α*; (3) inhibiting lipogenesis via downregulation of SREBP-1c by activating AMPK; and (4) enhancing antioxidant defense through Nrf2 pathway, as shown in Figure 2.

NF-*κ*B pathway regulates a variety of cytokines involved in inflammation. As a matter of fact, the anti-inflammatory effects of polyphenols have been generally subscribed to the inhibition of canonical NF-*κ*B pathway. The canonical NF-*κ*B pathway is activated by proinflammatory signals, causing the degradation of I*κ*B kinase (IKK) complex to release NF-*κ*B into the nucleus, with the consequence of inflammatory response [46]. It was reported that kaempferol inhibited the phosphorylation of insulin receptor substrate 1 (IRS-1), IKK*α*, and IKK*β*, accompanied with reduction of NF-*κ*B in nucleus and cytoplasm and further reduced TNF-*α* and IL-6 levels in mice with insulin resistance and type 2 diabetes mellitus [48]. Other polyphenols such as curcumin [49], morin [50], oligonol [51], and quercetin [52] also have been indicated to inhibit NF-*κ*B pathway in NAFLD. MAPK, a class of serine/threonine protein kinase widely expressed in mammalian cells including extracellular signal-regulated kinases (ERKs), JNK, and p38MAPK, is also closely associated with inflammation. Regulating MAPK, in particular with JNK and p38MAPK, has been regarded as the potential action mechanism of some polyphenols for the treatment of NAFLD. For example, cocoa flavonoids and apple polyphenols showed beneficial effects on redox balance and insulin resistance by targeting MAPKs in the context of NAFLD [53, 54].

PPAR, a group of nuclear receptors that play a role in lipid and glucose metabolism, is one of the promising targets in terms of regulating metabolic process [46]. Among the three types of PPARs that have been identified, PPAR*α* and PPAR*γ* have been highlighted for their involvement in the pathogenesis of NAFLD [47]. PPAR*α* is highly expressed in the liver to regulate free fatty acid (FFA) transport and stimulates enzymes participated in *β*-oxidation. Furthermore, it attenuates inflammation by inhibition of NF-*κ*B and C-reactive protein expression [46]. Therefore, stimulation of PPAR*α* is expected to relieve steatosis and hepatic inflammation. A great deal of study has demonstrated that many polyphenols can stimulate PPAR*α*. Some of them act as ligands and agonists of PPAR*α* [55, 56], while others, such as kaempferol [57], naringenin [58], tiliroside [59], and glabridin [60], can upregulate PPAR*α* gene and/or protein expression. Flavonoid-enriched extract from *Hippophae rhamnoides* seed decreases high-fat diet- (HFD-) induced obesity, hypertriglyceridemia, and hepatic triglyceride accumulation via regulation of PPAR*α* and PPAR*γ* gene expression and suppression of adipose tissue inflammation [61]. Compared with other drugs, such as glitazones, polyphenols have an advantage of partial activation of PPARs. This significantly reduces the risk of serious side effects by the use of full agonists, suggesting the great potency of polyphenols for the prevention and treatment of NAFLD. Another identified target in terms of metabolic regulation for the treatment of NAFLD is SREBP-1c, a transcription factor that regulates de novo lipogenesis through mediation of lipogenic enzymes and genes [46]. The increased expression of hepatic SREBP-1c promotes the progression of steatosis. A variety of polyphenols, such as genistein [62], luteolin [63], rutin [64], and prunetin [65], have been demonstrated to inhibit SREBP-1c, mainly via directly downregulation of SREBP-1c protein and gene expression, activation of AMPK, or inhibition of liver X receptor *α* (LXR*α*) that controls SREBP-1c transcription. In addition, as hyperinsulinemia stimulates SREBP-1c transcription, polyphenols might also decrease SREBP-1c by improving insulin sensitivity and controlling insulin levels. Furthermore, it has been proposed that polyphenols could inhibit SREBP-1c through inhibition of ER stress [66].

AMPK, a heterologous trimeric protein kinase that is formed by *α*, *β*, and *γ* subunits, is a vital regulator of cellular energy homeostasis [46]. It controls fatty acid metabolism through mediating the fatty acid biosynthetic pathway. In the pathogenesis of NAFLD, AMPK is closely related with insulin resistance and hepatic lipid accumulation [47]. AMPK activation inhibits the expression of ACC and FAS by downregulating SREBP-1c, thus reducing synthesis of fatty acids, cholesterol, and triglycerides and promoting fatty acid uptake and *β*-oxidation [46]. There is a great deal of polyphenols that serves as AMPK activators to protect hepatocytes against damage, such as resveratrol [67] and curcumin [49]. Hawthorn leaf flavonoids alleviated NAFLD by enhancing the adiponectin/AMPK pathway to regulate SREBP-1c, PPAR*α*, and related downstream targets [68]. Liquiritigenin protected hepatocytes against oxidative hepatic injury and mitochondrial dysfunction via AMPK activation by liver kinase B1 (LKB1) pathway as well as Farnesoid X receptor (FXR) induction [69].

#### 3.3.2. Other Emerging Mechanisms

MicroRNAs (miRNAs) are small noncoding RNAs that regulate gene expression at the posttranscriptional level. The role of miRNAs in NAFLD has been revealed and emphasized in recent years. A study measured circulating miRNAs in 84 nonalcoholic steatohepatitis (NASH) patients found that miR-122, miR-192, miR-19a, miR-19b, miR-125b, and miR-375 were significantly upregulated. Furthermore, the expression of miR-122, miR-192, and miR-375 correlated with disease severity. Increasing evidences claim that these circulating miRNAs not only serve as biomarkers for diagnosis but also play important roles in the intercellular communication and disease progression, which makes them attractive therapeutic targets. Exogenous factors such as polyphenols are suspected to affect miRNA concentrations to treat NAFLD. For example, miR-33 and miR-122, serving as major regulators of lipid metabolism in liver, were decreased in HFD-induced obese rats. It has been demonstrated that reduction of miR-122 induces IR, which can be reversed by licorice flavonoid [70]. Long-term supplementation with a low dose of proanthocyanidins could normalize liver miR-33a and miR-122 levels [71]. Plant-derived polyphenols were demonstrated to mediate the expression of miRNA paralogs miR-103/107 and miR-122 to attenuate NAFLD in hyperlipidemic mice [72]. Lychee pulp phenolics, mainly including quercetin 3-O-rutinoside-7-O-alpha-L-rhamnosidase (quercetin 3-rut-7-rha), rutin, and (−)-epicatechin, ameliorated liver lipid accumulation by reducing miR-33, which directly modulated adenosine triphosphate- (ATP-) binding cassette transporters ABCA1 and carnitine palmitoyltransferase 1 (CPT1) as well as miR-122 expression and indirectly regulated FAS, in mice with HFD [73].

Gut microbiota has been intensively researched due to its vital role in maintaining human health [74]. It is believed to be involved in obesity, metabolic syndrome, and the development of NAFLD [74]. A study has indicated that quercetin possessed ability of modulating intestinal microbiota imbalance and related gut-liver axis activation. Dysbiosis induced by HFD was accompanied by endotoxemia, intestinal barrier dysfunction, and gut-liver axis alteration, which could regulate TLR-4-NF-*κ*B signaling pathway activation, resulting in inflammasome initiation response and reticulum stress pathway induction [52]. Quercetin could revert gut microbiota imbalance and TLR-4 pathway induction, resulting in the blockage of deregulation of lipid metabolism genes. The striking benefits of polyphenols on NAFLD in mediating gut microbiota should be explored more in future studies, which might offer a new direction in understanding their action mechanisms.

The immune system is extensively implicated in the pathogenesis of NAFLD. Autophagy was recently identified as a critical protective mechanism during NAFLD development [75]. Lipophagy, defective autophagy of lipid droplets in hepatocytes, has recently been identified as a possible pathophysiological mechanism of NAFLD. Bergamot polyphenol fraction treatment (50 mg/kg/day supplemented with drinking water for 3 months) potently counteracted the increase of serum triglycerides, which was accompanied with increased levels of LC3 and Beclin 1 and reduced SQSTM1/p62, suggesting autophagy stimulation [76]. The development of a preventive treatment targeting circulating monocytes and hepatic macrophages as well as other immune cells such as CD4^+^ cells has been getting increased attention. Curcumin, possessing remarkable ability to prevent HFD-induced hepatic injury and metabolic derangements, was found to regulate intrahepatic CD4^+^ cell accumulation and inhibit inflammatory and oxidative damage caused by linoleic acid and leptin on mouse liver macrophages [77]. Activated macrophages/Kupffer cells promote the progression of hepatic fibrogenesis and aggravate metabolic disorders such as insulin resistance. Dietary quercetin supplementation to obesity mice decreased levels of TNF-*α* and IL-6, while it increased the level of anti-inflammatory cytokine IL-10 in the livers, accompanied by macrophage phenotype switching, as evidenced by upregulated anti-inflammatory M2 macrophage marker genes arginase 1 and Mannose receptor C (Mrc1), and downregulated proinflammatory M1 macrophage marker genes TNF-*α* and nitric oxide synthase 2 (NOS2). The beneficial effect of quercetin on NAFLD might be associated with promoting hepatic macrophage polarization in favor of the M2 phenotype via Nrf2-mediated heme oxygenase-1 (HO-1) induction [78].

Furthermore, accumulating evidence revealed the critical role of endoplasmic reticulum (ER) in NAFLD [47]. ER is the site of triglyceride synthesis and nascent lipid droplet formation with function in synthesizing, folding, and transporting proteins [47]. The accumulation of misfolding proteins in the ER lumen causes unfolded protein response (UPR) via the activation of the ER stress *sensor* proteins including PERK, inositol-requiring enzyme 1 (IRE1), and activating transcription factor 6 (ATF6). Sustained unfolded protein response (UPR) induces ER stress and metabolic disruptions, facilitating inflammation and insulin resistance in adipocytes. Lipolysis in response to ER stress is triggered via cAMP/protein kinase A (PKA) and ERK1/2 signaling. Curcumin treatment inhibited adipose tissue ER stress by dephosphorylation of inositol-requiring enzyme 1*α* and eukaryotic initiation factor 2*α* and reduced cAMP accumulation by preserving phosphodiesterase 3B induction, with the consequence of blockage of PKA/hormone-sensitive lipase lipolysis signaling, and thereby decreased glycerol and FFA release from adipose tissue [79]. Glycycoumarin, a representative of coumarin compounds isolated from licorice, showed inhibition of hepatocyte lipoapoptosis via suppressing ER stress-mediated JNK activation [80]. Furthermore, researches have reported that polyphenol extraction of grape [81] and its major bioactive compound resveratrol showed benefit for NAFLD partly through attenuating ER stress.

In addition, compelling evidence in recent years has demonstrated a significant link between NAFLD and cardiovascular disease (CVD) including coronary heart disease and stroke [82]. The likely of mechanisms underlying this association has been proposed involving genetic predisposition, insulin resistance, oxidative stress, chronic inflammation, atherogenic dyslipidemia, decreased adiponectin, and altered generation of pro- and anticoagulant factors [83]. In particular, among mechanisms linking CVD risk with hepatic steatosis, the most prominent factors are considered to be insulin resistance, chronic inflammation, oxidative stress, and atherogenic dyslipidemia [84]. The oxidative stress in NAFLD may induce alterations in endothelial function resulting in formation and deposition of oxidized low-density lipoprotein (LDL) in the subintimal space [82]. Therefore, therapeutic strategies targeting oxidative stress reduction in NAFLD patients for lowering CVD risk have been proposed. As an important category of antioxidants, polyphenols, such as resveratrol and silybin, have been attempted to reduce CVD risk in the setting of NAFLD [82]. Resveratrol, due to its potent effects on oxidative stress and inflammation, has become one of the most interesting candidates [84]. The effect of resveratrol on CVD protection has been demonstrated as evidenced by an improvement of CVD risk markers, such as endothelial function, echocardiographic parameters, and cytokine expression [85]. Studies, particularly long-term randomized clinical trials, evaluating the anticardiovascular effects of antioxidant treatment in patients with NAFLD are needed.

### 3.4. Viral Hepatitis

There are five well-characterized hepatotropic viruses, termed hepatitis A to hepatitis E. Among them, hepatitis B virus (HBV) and hepatitis C virus (HCV) are the most common types. In particular, HBV is a major cause of liver cirrhosis and HCC [86]. Though there is opportunity to prevent and treat viral hepatitis, all the currently approved antiviral drugs have their limitations [86]. For example, interferon has limited efficacy with a high incidence of adverse effects in some patients. As an alternative approach, natural products have provided great promises as potentially effective antiviral drugs. A broad spectrum of phytochemicals including flavonoids such as wogonin and polyphenolics such as geraniin has been isolated and investigated for antihepatitis virus activities in vitro as well as in vivo [87, 88]. The underlying action mechanisms have been proposed mainly as prevention of virus entry, inhibition of viral antigen secretion, and suppression of DNA replication [89].

Several flavonoids have been identified as inhibitor of HCV and HBV entry. A potent inhibitor of hepatitis virus is EGCG, a well-known polyphenol in green tea. EGCG inhibited entry of HBV into hepatocytes via induction of clathrin-dependent endocytosis of sodium taurocholate cotransporting polypeptide from the plasma membrane followed by protein degradation and inhibited the clathrin-mediated endocytosis of transferrin, without effect on HBV genome replication or virion secretion [90]. It can also potently inhibit HCV entry into hepatoma cell lines and primary human hepatocytes [91, 92]. Delphinidin, a plant pigment in flavonoid family that is responsible for the blue-purple color of flowers and berries, induced a bulging of the viral envelope to inhibit HCV attachment to the cell surface [93]. Tannic acid could inhibit HCV entry into Huh7.5 cells [94].

Plenty of studies have reported polyphenols with remarkable antihepatitis virus through inhibiting virus replication via different mechanisms. Silibinin served as direct inhibitor of HCV RNA-dependent RNA polymerase [95]. Epicatechins, one of the phenolic in green tea, can inhibit HCV replication via cycloxygenase-2 and relieve inflammation induced by virus [96]. The flavonoid apigenin inhibited HCV replication by decreasing mature miRNA122 levels, which was a liver-specific miRNA for positive regulation of HCV replication [97]. Curcumin suppressed HBV via downregulation of the metabolic coactivator PGC-1*α*, a starvation-induced protein that has been shown to robustly coactivate HBV transcription [98]. The flavonoid prescription baicalin-linarin-icariin-notoginsenoside R1 had curative effect on duck virus hepatitis caused by duck hepatitis A virus type 1 (DHAV-1), which could inhibit DHAV-1 reproduction by destroying its adsorption and release [99]. Quercetin significantly reduced the viral genome replication, the production of infectious HCV particles, and the specific infectivity of the newly produced viral particles [100]. Nonstructural protein 3 (NS3) encoded by HCV genome has been regarded as a potential anti-HCV drug target as it is vital for viral replication. Several anthracyclines with hydroxyanthraquinone moiety structure were found to inhibit NS3 helicase. And mitoxantrone, a hydroxyanthraquinone analogue, was also known to be inhibitor of NS3 helicase [101]. Additionally, quercetin suppressed HCV via inhibition of NS3 protease activity [102]. Another target for HCV, nonstructural protein 5B (NS5B), could be inhibited by EGCG [103]. Other compounds showing antiviral activities including kaempferol 8-methyl ether, quercetin 3-methyl ether, kaempferol [104], chlorogenic acid analogues, isoliquiritigenin, glycycoumarin, [105], glycyrin, glycyrol, liquiritigenin, isoliquiritigenin, licochalcone A, and glabridin chlorogenic acid analogues [106] identified from natural products such as tea and medicinal plants would also be good candidates for development of antivirals against hepatitis virus.

### 3.5. Liver Fibrosis and Cirrhosis

**Figure 3:**
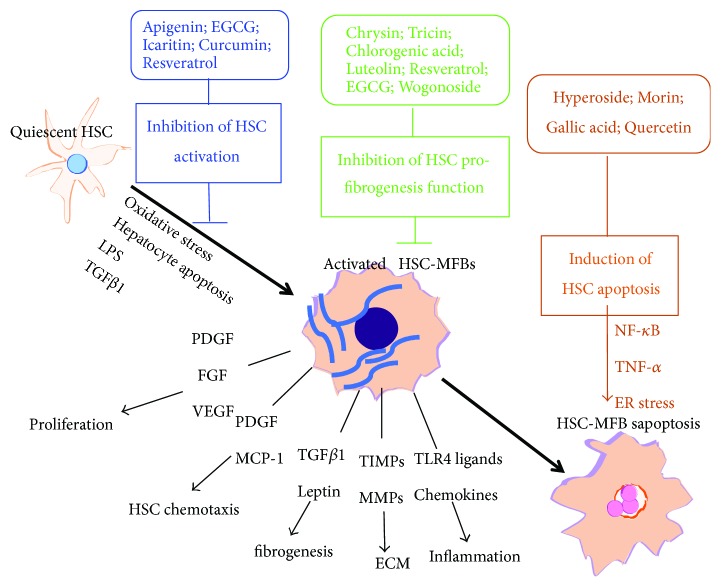
The major action mechanisms of a variety of polyphenols on HSCs. Apigenin, EGCG, icaritin, curcumin, and resveratrol could inhibit the activation of HSCs; chrysin, tricin, chlorogenic acid, luteolin, resveratrol, EGCG, and wogonoside suppress the profibrogenesis function of HSCs; hyperoside, morin, gallic acid, and quercetin can induce HSC apoptosis. PDGF: platelet-derived growth factor*;* FGF: fibroblast growth factor; MCP-1: monocyte chemoattractant protein-1; TIMP1: TIMP metallopeptidase inhibitor 1.

Liver fibrosis is a wound-healing response to hepatic injury. It is characterized by the accumulation of extracellular matrix (ECM), which leads to a progressive substitution of liver parenchyma by scar tissue [3]. Sustained fibrogenesis would result in cirrhosis, the consequence of progressive fibrosis with a poor outcome and high mortality, characterized by a distortion of the liver parenchyma and vascular architecture. Due to the vital role of ECM in fibrogenesis, matrix-expressing cells have been considered as the vital cellular basis of liver fibrogenesis [3]. Among them, hepatic stellate cells (HSCs), the major cell type responsible for ECM deposition, have been extensively studied. Upon chronic liver injury, quiescent HSCs undergo morphological and phenotypical transdifferentiation into contractile and highly proliferative myofibroblasts with collagen-producing ability. Plenty of polyphenols were found to protect liver from fibrosis via suppression of the activation of HSCs such as apigenin, EGCG, quercetin, icaritin, curcumin, and resveratrol [107–113]. The mechanisms underlying the inhibition of activated HSCs have been ascribed to upregulation of C1QTNF2, MMPs, or miR-221 to accelerate osteopontin degradation and downregulation of PPAR*γ* or membrane translocation and gene expression of GLUT2. Polyphenols such as hyperoside, morin, gallic acid, and quercetin have been revealed that they exert antifibrosis effect via promoting apoptosis of activated HSCs, primarily associated with NF-*κ*B and TNF-*α* signaling [114, 115]. Furthermore, there is mounting evidence indicated that many flavonoids could decrease proliferation of HSCs and inhibit expression of profibrogenesis-related genes in HSCs. For example, chrysin and tricin inhibited proliferation of HSCs via suppressing TGF-*β*1/Smad pathway and blocking tyrosine phosphorylation of platelet-derived growth factor (PDGF) receptor, respectively [116, 117]. Chlorogenic acid suppressed profibrotic action of HSCs via inhibition of NOX/ROS/MAPK pathway, while resveratrol can suppress the activation of NF-*κ*B and Akt, reducing expression of related profibrogenesis genes in activated HSCs [118, 119]. EGCG and wogonoside regulate profibrogenic/antifibrogenic balance via inhibition of PI3K/Akt/Smad pathway and PI3K/Akt/mTOR/ribosomal protein S6 kinase 70 kDa (p70S6K), respectively [120, 121]. We summarized the major action mechanisms of a variety of polyphenols on HSCs in Figure 3. In addition to action on HSCs, several polyphenols such as morin, chlorogenic acid, and curcumin also showed antifibrotic capacities through attenuating oxidative stress and inflammation response via different mechanisms including inhibition of TLR4/MyD88/NF-*κ*B signaling pathway, suppressing the advanced glycation end- (AGE-) mediated induction of receptor for advanced glycation end (RAGE) gene expression by increasing PPAR*γ* and stimulating glutathione (GSH), and regulating PPAR signal pathway and the interaction with FXR [122–124]. In summary, the potential mechanisms of some polyphenols with remarkable antifibrotic ability *in vivo* and *in vitro* have been listed in Table 2. The promising antifibrotic efficiencies of these compounds from food and plants with well-recognized action mechanisms make them deserve further exploration in a clinical study in the future.

### 3.6. Liver Cancer

Liver cancer, the sixth most common cancer with high mortality worldwide, represents a major international health problem. Increasing epidemiological evidence indicated that a diet rich in fruits and vegetables could lower the risk of certain cancers, including liver cancer, which has been partly attributed to natural polyphenols contained [128]. A variety of natural polyphenols have been studied for the prevention and treatment of liver cancer [129]. Potential mechanisms have been proposed as proapoptosis and antiproliferation effect to liver cancer cells, antiangiogenesis, inhibition of invasion, and metastasis, as well as other modulation of multiple molecular events involved in carcinogenesis.

A plenty of polyphenols showed remarkable properties of promoting apoptosis and suppressing proliferation of liver cancer cells via various pathways [130–133]. Many flavones, a class of flavonoids based on the backbone of 2-phenylchromen-4-one, such as vitexin, luteolin, chrysin, isoorientin, oroxylin A, wogonin, and baicalein, have been found to induce apoptosis and inhibit proliferation of a variety of HCC cell lines by different or overlapped mechanisms. UPR pathway, mTOR pathway, ROS pathway, JNK pathways, caspase-dependent, and caspase-independent apoptotic signaling pathways have been extensively proposed for anticancer ability of these flavones. Flavanones including eriodictyol and hesperidin induce HepG2 cell apoptosis mainly via regulation of apoptotic proteins such as Bax and Bcl-2, mitochondrial pathway, and death receptor pathway. Other flavonoids including flavanols, flavanonol, flavonol, and isoflavones, which have been demonstrated to induce apoptosis of liver cancer cells *in vivo* and *in vitro*, are summarized in Table 3. Nonflavonoids, such as gigantol, chlorogenic acid, and gallic acid, mediate the apoptosis of HCC cells principally through induction of ER stress and regulating mitochondrial-mediated pathways. As a matter of fact, most polyphenols induce apoptosis and inhibit proliferation of HCC cells via multiple targets and pathways. For example, baicalein caused HepG2 cell apoptosis via inhibiting the PKB/mTOR pathway or blocking MEK-ERK signaling [134, 135] while another flavone compound oroxylin A suppressed PI3K-PTEN-Akt-mTOR signaling pathway and activated the PERK-eIF2*α*-ATF4-CHOP branch of the UPR pathway [136, 137] to mediate apoptosis. EGCG, a famous flavanol in tea, showed remarkable ability to induce apoptosis of a variety of liver cancer cells such as SMMC7721, SK-hep1, HLE, HepG2, HuH-7, and PLC/PPF/5 cells [132]. Its underlying mechanisms have been revealed as inhibition of receptor tyrosine kinase, downregulation of PI3K/AKT activity, downregulation of Bcl-2*α*, and Bcl-xl by inactivation of NF-*κ*B [138–140]. Additionally, fisetin, a common flavonoid found in many fruits and vegetables, suppressed proliferation of liver cancer cells via modulation of multiple signaling pathways including CDK5 signaling, Nrf2-mediated oxidative stress response, glucocorticoid signaling, and ERK/MAPK signaling [141]. The character of modulation of multiple targets and pathways makes them more promising in application of developing anticancer drugs or dietary supplements for HCC patients.

Furthermore, several flavonoids have been demonstrated to exert beneficial effects against liver cancer via antiangiogenesis. Flavones including eupafolin and morusin showed significant antiangiogenic abilities by *in vitro* and *in vivo* studies. Eupafolin could block vascular endothelial growth factor- (VEGF-) induced activation of vascular endothelial growth factor receptor 2 (VEGFR2) in Akt activity in human umbilical vascular endothelial cells and inhibit Akt activity and VEGF secretion in HepG2 [142]. The other flavone, morusin, inhibited angiogenesis in HepG2 xenograft mice model via attenuation of the IL-6 and signal transducer and activator of transcription 3 (STAT3) signaling pathway [143]. Morin, a type of flavonol belonging to flavonoid, inhibits tumor growth and angiogenesis in rats with diethylnitrosamine- (DEN-) induced HCC through upregulation of NF-*κ*B-p65 and COX-2 and reducing MMPs [144]. Resveratrol also showed effects on suppressing angiogenesis in mice with HCC xenograft. The mechanism underlying its antiangiogenesis is through inhibiting VEGF expression by a NF-*κ*B-mediated pathway [145].

In addition to antiangiogenesis, increasing studies indicated that a variety of polyphenols could inhibit invasion and metastasis in liver cancer. Flavanones including hesperidin and naringenin, flavones including luteoloside and wogonin, and other flavonoids such as galangin, EGCG, and genistein have been reported by various studies for reducing invasion and metastasis of liver cancer cells *in vivo* and *in vitro*. It is interesting to note that hesperidin and naringenin, two types of flavanone, inhibited invasion and metastasis of liver cancer cells such as HepG2 via similar mechanisms, reducing MMP-9 expression through the inhibition of NF-*κ*B and activator protein 1 (AP-1) activity, suggesting potential structure-activity relationship might exist between flavanone and NF-*κ*B/AP-1 pathways. Additionally, phenolic compounds, theaflavins and (−)-oleocanthal suppressed the growth and metastasis through the blockage of STAT3 pathway. Their structure-activity relationship deserves to be further explored in the future. Other flavonoids possessing anti-invasion or antimetastasis effects by various pathways in HCC have also been summarized in Table 3.

Recently, several polyphenols have been demonstrated to reduce carcinogenesis. Curcumin treatment effectively reduced the progression of NASH to HCC by suppressing the protein expression of glypican-3, VEGF, and prothrombin in the NASH liver [86]. The upregulation of self-renewal Wnt/*β*-catenin, Hh/Gli1 pathways, and their associated genes cyclin D1, cMyc, and epidermal growth factor receptor (EGFR) along with downregulation of E-cadherin during the carcinogenesis processes was found to be modulated by EGCG/theaflavins [146, 147]. In addition, some emerging pathways have been proposed to be involved in mechanisms of polyphenols' anticancer effect. Glycycoumarin exerts antiliver cancer activity by directly targeting oncogenic kinase T-LAK cell-originated protein kinase (TOPK) [148]. Isoorientin possessed a notable hepatoprotective effect in the context of liver cancer, which might be mediated through the respiratory chain complexes and phase II detoxifying enzyme activities [149]. Flavonoids activated pregnane X receptor-mediated CYP3A4 gene expression by inhibiting cyclin-dependent kinases in HepG2 liver carcinoma cells [150]. Furthermore, chlorogenic acid and catechins have been demonstrated to augment the antitumor effect of chemotherapeutic drugs for liver cancer. Chlorogenic acid could sensitize HCC cells to 5-fluorouracil treatment by inhibiting ERK activation through the overproduction of ROS [151]. Catechins enhanced the antitumor activity of doxorubicin for liver cancer involving the suppression of multidrug resistance protein 1 (MDR1) expression or accumulation increase of intracellular doxorubicin [152].

## 4. Clinical Studies

As *in vitro* and *in vivo* animal studies have revealed the promising preventive and therapeutic effects of polyphenols in various liver diseases, translational studies are extremely vital and indispensable for the application of polyphenols in human with liver diseases. Although literatures in PubMed database about clinical trials of polyphenols in liver diseases are limited, encouraging beneficial effects of these polyphenols have been demonstrated, particularly in NAFLD. In a compliant, randomized, double-blind, placebo-controlled pilot trial of purified anthocyanin in NAFLD patients, supplementation of purified anthocyanin for 12 weeks significantly improved insulin resistance, liver injury, and clinical evolution in those patients [193]. In another double-blind clinical trial, dihydromyricetin, the main active ingredient of *Ampelopsis grossedentata*, improved glucose and lipid metabolism and showed anti-inflammatory effects in NAFLD [194]. Intervention with green tea having high-density catechins significantly enhanced liver function and fat infiltration in NAFLD patients in a randomized double-blind study [195]. In particular, resveratrol, which showed extraordinary benefit for NAFLD in animal studies, has been attempted by several clinical trials. In a double-blind, randomized and placebo-controlled study, treatment with 2150 mg resveratrol capsules twice daily for three months significantly reduced the levels of TNF-*α*, cytokeratin 18 fragment, and fibroblast growth factor 21 (FGF-21) and improved adiponectin level in NAFLD patients, suggesting its beneficial role in NAFLD [196]. In another study, intervention with resveratrol at the dose of 1000 mg daily for week 1 followed by 2000 mg daily for week 2 significantly reduced intestinal and hepatic lipoprotein particle production in overweight/obese men [197]. However, a study claimed that resveratrol showed no benefit for patients with NAFLD. It might be due to the dosage used or intervention duration was not enough for resveratrol to exert hepatoprotective effects [198]. Additionally, intervention of orange juice with abundant flavonoids for HCV patients showed lower levels of total cholesterol and LDL-cholesterol and increased antioxidant capacity compared to that of the control group [199].

To learn about currently ongoing and unpublished studies, we further looked up the related information of clinical trials of a variety of representative polyphenols in liver diseases at the database of U.S. National Library of Medicine (http://www.ClinicalTrials.gov). The polyphenols searched are anthocyanins including delphinidin, pelargonidin, cyanidin, and malvidin; flavanols including epicatechin, epigallocatechin, EGCG, and procyanidins; flavanones including hesperidin and naringenin; flavones including apigenin, chrysin, luteolin, oroxylin A, wogonin baicalein, and isoorientin; flavonols including quercetin, kaempferol, myricetin, isorhamnetin, and galangin; isoflavonoids including genistein and daidzein; phenolic acids including ellagic acid, gallic acid, ferulic acid, and chlorogenic acid; other polyphenols including curcumin, sesamin, secoisolariciresinol diglucoside; resveratrol; pterostilbene; and piceatannol. Among them, only several compounds including curcumin, chlorogenic acid, resveratrol, EGCG, quercetin, naringenin, and catechin are found to be studied in NAFLD, HCV, cirrhosis, and liver cancer, which are listed in Table 4. Other polyphenols, which showed promising therapeutic effects on liver diseases in animal studies, such as baicalein, wogonin, kaempferol, and theaflavins, deserve to be translationally studied by clinical trials in the future.

## 5. Conclusions and Prospects

As multitude of pathways is involved in the pathogenesis of liver diseases, therapies targeting multiple factors are expected to address these driving forces for liver disease progression. Natural polyphenols, widely existing in plants and plant-based food, have attracted increasing attention as potential agents for prevention and treatment of liver diseases due to their outstanding effects on mediating pathways involved in the pathogenic process. As a matter of fact, the multiple regulations on oxidative stress, ER stress, inflammation, immune response, lipid metabolism, insulin resistance, and gut microbiota by various polyphenols are the scientific fundaments for the application of polyphenols in the prevention and treatment of liver diseases. However, although therapy by polyphenols for liver diseases has been proposed for decades and encouraging efficiency has been obtained by *in vitro* and *in vivo* studies, there is still a long way to go for the use of polyphenols in human. Several difficulties in translational research are challenging ahead. For those studies in which dose effect has been investigated, only certain polyphenols showed dose-effect manner for attenuating liver injury, suggesting the importance of determination of optimized dosage to be used. The route of administration is also a vital factor for absorption and bioavailability of polyphenols. Actually, one of the key limitations relating to the use of polyphenols is their poor bioavailability. Improving bioavailability via modification of delivery route or administration route is of great importance for translational study of polyphenols. In addition, as certain polyphenols may have side effects such as carcinogenic/genotoxic effects or disordering thyroid hormone biosynthesis, risks and safety of polyphenol consumption in liver diseases should also be well noted. It is of great importance to evaluate the doses at which these effects occur. Therefore, future studies evaluating either beneficial or adverse effects including relevant forms and doses of polyphenols should be performed. More importantly, further clinical trials are needed to evaluate the exact effects of a variety of polyphenols in patients with liver diseases, particularly for those showing remarkable therapeutic efficiency in animal study. In conclusion, a great deal of flavonoid and phenols has been demonstrated to exert multifaceted actions on various liver diseases by well-recognized mechanism, indicating their great potential in the prevention and treatment for liver diseases. In future study, the effective and safe dose, duration of treatment, absorption and bioavailability of polyphenols should be thoroughly investigated from benchtop and bedside.

## Figures and Tables

**Table 1:** The representative species with relatively high quantities of polyphenols in foods and plants.

Names	Content of polyphenols (mg GAE/g)	References
*Vegetables*		
Chinese toon bud	23.27	[11]
Perilla leaf	14.37	
Loosestrife	13.13	
Soybean (green)	12.39	
Pepper leaf	12.14	
*Fruits*		
Chinese date	5.86	[12]
Sweetsop	4.05	
Guava	1.94	
Pomegranate	1.47	
Chinese wampee	1.16	
*Cereals*		
Black rice	9.47	[13]
Organic black rice	6.95	
Purple rice	4.85	
Buckwheat	4.48	
Red rice	4.43	
*Spices*		
Clove	143.8	[14]
Cinnamon stick	119.0	
Oregano	101.7	
Cinnamon	63.4	
Sage	53.2	
*Mushrooms*		
*Thelephora ganbajun* zang	44.84	[15]
*Boletus edulis* Bull	14.15	
*Volvariella volvacea* Sing	13.91	
*Boletus regius* Krombh	10.17	
*Suillus bovinus* Kuntze	9.19	
*Tea*		
Fu'andabai	223.7	[16]
Shuyong number 1	221.6	
Sichuanxiaoye	215.0	
Shuyong number 2	215.0	
Menghaidayi	215.0	
*Microalgae*		
*Nostoc ellipsosporum* CCAP 1453/17	60.35	[17]
*Chlorella protothecoides* number 7	19.03	
*Chlorella pyrenoidosa* number 3	17.24	
*Schizochytrium* sp. number 5	15.94	
*Chlorella pyrenoidosa* number 2	15.11	
*Medical plants*		
*Salvia miltiorrhiza* Bge	101.33	[18]
*Sargentodoxa cuneata* Rehd et Wils	65.28	
*Prunus persica* (Linn) Batsch	55.23	
*Fraxinus rhynchophylla* Hance	52.31	
*Rhodiola sacra* Fu	51.06	
*Picrorhiza scrophulariflora* Pennell	47.28	
*Scutellaria baicalensis* Ceorgi	46.31	
*Polygonum multiflorum* Thunb (stem)	45.24	
*Tussilago farfara* L.	34.50	
*Polygonum multiflorum* Thunb (root)	31.87	
*Wild fruits*		
*Eucalyptus robusta*	54.8	[19]
*Eurya nitida*	35.0	
*Melaleuca leucadendron*	25.6	
*Gordonia axillaris*	24.6	
*Melastoma sanguineum*	23.3	
*Edible and wild flowers*		
*Rosa hybrida*	35.84	[20]
*Limonium sinuatum*	34.17	
*Pelargonium hortorum*	25.68	
*Jatropha integerrima*	17.22	
*Osmanthus fragrans*	16.00	
*Fruit wastes*		
Grape seed	22.95	[21]
Mango peel	22.95	
Sweetsop peel	17.77	
Longan seed	13.58	
Chinese olive peel	13.16	

**Table 2:** The potential antifibrotic mechanisms of some polyphenols.

Effects	Polyphenol	Model	Mechanisms	Ref.
Inhibition of HSC activation	Apigenin	*In vitro* HSCs	Upregulating C1QTNF2 expression	[107]
	EGCG	*In vitro* HSCs & thioacetamide-treated animal	Upregulating miR-221 to accelerate osteopontin degradation	[108]
	Quercetin	Rats with CCl4-induced fibrosis	Activation of MMPs and regulating profibrogenic/antifibrogenic molecules balance	[109]
	Icaritin	*In vitro* HSC-T6 and LX-2 HSC lines & rats with CCl4 or CBDL-induced fibrosis	Dependent on mitochondrial-activated apoptosis	[110]
	Curcumin	*In vitro* HSCs & animal model	Suppressing membrane translocation and gene expression of GLUT2; inhibiting PPAR*γ* via regulation of DLK1 protein partly mediated by interruption of Shh signaling pathway	[111, 112]
	Resveratrol	Rats with N′-nitrosodimethylamine-induced liver fibrosis	Relieving oxidative damage	[113]

Induce HSC apoptosis	Hyperoside	*In vitro* human LX-2 HSCs	Inhibiting the DNA-binding activity of NF-*κ*B and altered genes related to apoptosis	[125]
	Morin	*In vitro* HSCs	Suppressing canonical NF-*κ*B signaling	
	Gallic acid	*In vitro* HSCs	Regulating TNF-*α* signaling pathway	[114]
	Quercetin	*In vitro* HSCs	Dependent on activation of ER stress	[115]

Inhibit proliferation and profibrogenesis-related genes in HSCs	Chrysin	Mice with CCl4-induced fibrosis	Suppressing TGF-*β*1/Smad pathway	[116]
	Tricin	*In vitro* human HSC line LI90	Blocking tyrosine phosphorylation of PDGF receptor	[117]
	Chlorogenic acid	*In vitro* HSCs & CCl4-treated rats	Improving antioxidant capacity via activation of Nrf2 pathway and suppressing profibrotic action via inhibition of NOX/ROS/MAPK pathway	[118]
	Luteolin	*In vitro* rat HSCs and HSC-T6 cells & rat models induced by CCl4, DMN, and BDL	Increasing caspase 3 activity and p53 expression; inducing G1 arrest with the decreased expression of bcl-2, cyclin E, and p-Cdk-2; suppressing PDGF and TGF1-simulated phosphorylation of AKT and Smad pathway	[126]
	Resveratrol	*In vitro* human LX-2 HSCs & mice with CCl4-induced fibrosis	Suppressing the activation of NF-*κ*B and Akt	[119]
	EGCG	*In vitro* HSC LX-2 & BDL rats	Inhibiting PI3K/Akt/Smad pathway	[120]
	Wogonoside	*In vitro* HSC T6 cells & mice with CCl4-induced fibrosis	Inhibiting PI3K/Akt/mTOR/p70S6K	[121]

Attenuate liver injury and antifibrosis	Morin	Rats with CCl4-induced fibrosis	Reducing oxidative stress, inflammatory responses, and fibrogenic markers	[122]
	Chlorogenic acid	Rats with CCl4-induced fibrosis	Inhibition of TLR4/MyD88/NF-*κ*B signaling pathway	[123]
	Curcumin	Mice with type 2 diabetes mellitus	Suppressing the AGEs-mediated induction of RAGE gene expression by increasing PPAR*γ* and stimulating GSH	[124]
	Total flavonoids of *Astmgali Radix*	Rats with fibrosis	Regulating PPAR signal pathway and the interaction with FXR	[127]

**Table 3:** The effects and underlying mechanisms of polyphenols against liver cancer.

Classification	Polyphenols	Natural sources	Cell types/animal models	Effects	Involved mechanisms	Ref.
*Induce apoptosis and inhibit proliferation*						
Flavone	Vitexin	*Vitex agnus-castus*	SK-Hep1 and Hepa1-6 cells	Induce apoptosis	Activation of the JNK signaling pathway	[153]
	Luteolin	Celery, green pepper, parsley, thyme, dandelion, and others	HepG2 cells	Induce apoptosis	ROS-mediated pathway, regulating intrinsic and extrinsic caspases as well as executioner caspases	[154]
	Chrysin	Honey, propolis, the passion flowers, and *Passiflora caerulea*	HepG2 cells and QGY7701 cells	Reduce proliferation and cell motility as well as induce apoptosis	Downregulation of Skp2 and LRP6 expression; activation of the p53/Bcl-2/caspase-9 pathway	[155]
	Isoorientin	Passion flower, *Vitex negundo*, *Terminalia myriocarpa*	HepG2 cells	Induce apoptosis	Mitochondrial-mediated pathway: the regulation of cell cycle-related genes; elevate ROS formation, followed by attenuation of mitochondria membrane potential; increase in caspase-3 and caspase-9 proteolytic activities	[130]
	Luteolin-7-O-glucoside	Dandelion coffee and in *Cynara scolymus*	HepG2 cells	Induce apoptosis and inhibit proliferation	G2/M phase cell cycle arrest by JNK activation and caspase-independent apoptotic signaling pathways	[156]
	Oroxylin A	*Scutellaria baicalensis* and the Oroxylum indicum tree	HepG2 cells	Induce apoptosis	Suppressing of PI3K-FTEN-Akt-mTOR signaling pathway; activation of the PERK-eIF2*α*-ATF4-CHOP branch of the UPR pathway	[136, 137]
	Wogonin	*Scutellaria baicalensis*	HepG2, SMMC-7721, and Hep3B cells	Induce apoptosis and necrosis	Activation of the UPR pathway and consequent inactivation of AKT signaling	[157]
	Baicalein	Roots of *Scutellaria baicalensis* and *Scutellaria lateriflora*	HepG2 cells; HCC a in mice	Induce apoptosis and inhibit tumor growth	Inhibiting the PKB/mTOR pathway; blocking MEK-ERK signaling	[134, 135]

Flavanone	Eriodictyol	*Eriodictyon californicum*	HepG2 cells	Induce apoptosis	Upregulation of Bax and PARP and downregulation of Bcl-2 protein	[158]
	Hesperidin	Citrus fruits	HepG2 cells; xenograft tumors	Induce apoptosis	Regulating mitochondrial pathway and death receptor pathway; triggering the activation of the mitochondrial pathway by increasing the levels of intracellular ROS, ATP, and Ca^2+^.	[159]; [160]

Isoflavones	Puerarin	Root of Pueraria	SMMC-7721 HCC cells	Induce apoptosis	Regulating MAPK pathways	[161]

Flavonols	Galangin	*Alpinia officinarum* and *Helichrysum aureonitens*	HepG2, Hep3B, and PLC/PRF/5 cells	Induce apoptosis	Via mitochondrial pathway, translocating the proapoptotic protein Bax to the mitochondria to release apoptosis-inducing factor and cytochrome c into the cytosol; regulating MAPK signaling pathways	[162, 163]
	Kaempferol	Delphinium, grapefruit	HepG2 and Huh7 cells	Autophagy-mediated cell death	ER stress-CHOP-autophagy signaling pathway	[99]

Flavanols	EGCG	Tea	SMMC7721, SK-hep1, HLE, HepG2, HuH-7, and PLC/PPF/5 cells; a xenograft model	Induce apoptosis and antiproliferation	Inhibit receptor tyrosine kinase; downregulating PI3K/AKT activity; downregulating Bcl-2 alpha and Bcl-xl by inactivation of NF-*κ*B	[138, 139]

Flavanonols	Dihydromyricetin	Ampelopsis species japonica; *Hovenia dulcis*	HepG2 cells	Inhibit proliferation and induce apoptosis	Via a p53-dependent manner; reducing TGF-*β* via p53-dependent signal pathway	[164]

Other flavonoids	Daphnegiravone D	Daphne giraldii	Hep3B and HepG2; nude mouse xenograft model	Inhibit proliferation	Regulating p38 and JNK MAPK pathways	[165]
	Kurarinol	Roots of the medical plant *Sophora flavescens*	HepG2, Huh-7, and H22 cells; H22 tumor-bearing mice	Induce apoptosis	Suppressing STAT3 signaling	[166]
	Eriocitrin	Lemons	HCC cell lines	Induce apoptosis and arrest cell cycle	Arresting cell cycle in S phase through upregulation of p53, cyclin A, cyclin D3, and CDK6; trigger apoptosis by activating mitochondria-involved intrinsic signaling pathway	[167]
	Isoquercitrin	*Mangifera indica* (mango) and Rheum nobile (the Noble rhubarb)	Liver cancer cells; tumor-bearing nude mice	Induce apoptosis and inhibit tumor growth	Regulating MAPK and PKC signaling pathways	[168]
	Fisetin	Strawberries, apples, persimmons, onions, and cucumbers	Liver cancer cells	Induce apoptosis	Regulating CDK5 signaling, NRF2-mediated oxidative stress response, glucocorticoid signaling, and ERK/MAPK signaling	[141]

Nonflavonoids	Gigantol	Plants in the genus dendrobium	HepG2 cells	Inhibit proliferation	Regulating PI3K/Akt/NF-*κ*B signaling pathway	[169]
	Licochalcone A	Root of *Glycyrrhiza glabra* and Glycyrrhiza inflata	HepG2 cells	Induce apoptosis	Induction of ER stress via phospholipase C γ1 (PLC *γ*1), Ca^2+^, and ROS-dependent pathway	[170]
	3-decylcatechol	Sap of the lacquer tree	Huh7 cells	Autophagy-mediated cell death	Activating ER stress to promote autophagy via p62 transcriptional activation involving IRE1*α*/JNK pathways	[171]
	Curcumin	Ginger family	SMMC-7721 cells	Inhibit proliferation	Regulating AMPK signaling pathway	[172]
	Sesamol	Sesame seeds and sesame oil	HepG2 cells; a xenograft nude mice model	Suppress colony formation, inhibit the proliferation and promote apoptosis	Impairing mitochondrial function and suppressing autophagy through impeding the PI3K class III/Belin-1 pathway	[173]
	E-[6-(5-hydroxypentyl)tricosyl]-4-hydroxy-3-methoxycinnamate	Fruits of *Livistona chinensis*	HepG2 cells	Autophagy-related apoptosis; suppress cell proliferation and colony formation	Via a mitochondria-dependent caspase pathway in HepG2; induce autophagy via inhibition of the Akt/mechanistic target of rapamycin/p70 ribosomal protein S6 kinase signaling pathway	[174]
	Chlorogenic acid	Leaves of *Hibiscus sabdariffa*, eggplants, peaches, and prunes	HepG2 cells; HepG2 xenograft animal model	Inhibit proliferation and the progression of HepG2 xenograft	Inactivation of ERK1/2 and suppressed the expression of MMP-2 and MMP-9	[175]
	Gallic acid	Gallnuts, sumac, witch hazel, tea leaves, oak bark, and other plants	HepG2 and SMMC-7721 cells; DEN-induced HCC	Induce apoptosis and antiproliferation	Regulating mitochondrial-mediated pathways, induce caspase-3, caspase-9, and ROS activity, elevate Bcl-2-like protein 4, and reduce the mitochondrial membrane potential; decreasing the levels of argyophillic nucleolar organizing regions, and proliferating cell nuclear antigen	[176]

*Antiangiogenesis*						
Flavone	Eupafolin	*Artemisia princeps* Pampanini	Human umbilical vascular endothelial cells (HUVECs); HepG2	Antiangiogenesis	Blocking VEGF-induced activation of VEGFR2 in Akt activity in HUVECs; inhibiting Akt activity and VEGF secretion in HepG2	[142]
	Morusin	Root bark of *Morus alba*	HepG2 and Hep3B; HepG2 xenografts	Apoptosis induction and antiangiogenesis	Attenuation of the IL-6/STAT3 signaling pathway	[143]

Flavonol	Morin	*Maclura pomifera*, *Maclura tinctoria*, and from leaves of *Psidium guajava*	Rats with DEN-induced HCC	Antiangiogenesis	Upregulation of NF-*κ*B-p65 and COX-2; reducing MMP-2 and MMP-9	[144]

Flavonoid	Hydroxysafflor yellow A	*Carthamus tinctorius* L.	H22 tumor-bearing mice	Antiangiogenesis	Blocking ERK1/2 phosphorylation and then restraining the activation of NF-*κ*B, suppressing mRNA expression levels of cell proliferation-related genes cyclin D1, cMyc, and c-Fos	[177]

Nonflavonoids	Resveratrol	Grapes, berries, red wine	HCC xenograft animal model	Antiangiogenesis	Inhibiting VEGF expression through a NF-*κ*B-mediated mechanism	[145]

*Inhibit the invasion and metastasis*						
Flavanones	Hesperidin	Citrus fruits	HepG2 cells	Inhibit invasion and metastasis	Reducing MMP-9 expression through the inhibition of activated NF-*κ*B and AP-1 activity by I *κ*B, JNK, and p38 signaling pathways	[178, 179]
	Naringenin	Citrus fruits	HepG2, Huh-7, and HA22T cells	Inhibit the invasion and metastasis	Suppressing MMP-9 transcription by inhibiting NF-*κ*B and AP-1 activity	[180]

Flavone	Luteoloside	*Gentiana macrophylla*	HCC cells; mouse lung metastasis model	Suppress proliferation and metastasis	Inhibition of NLRP3 inflammasome	[181]
	Wogonin	*Scutellaria baicalensis*	HepG2 and Bel7402 HCC cells	Inhibit proliferation and invasion	Regulating NF-*κ*B/Bcl-2, EGFR, and EGFR downstream ERK/AKT signaling	[182]

Flavonol	Galangin	*Alpinia officinarum* and *Helichrysum aureonitens*	HepG2 cells	Inhibit metastasis	Protein kinase C (PKC)/ERK signaling pathway	[183]

Flavanols	EGCG	Tea	MHCC-97H and HepG2 cells	Inhibit metastasis	Reduce osteopontin by decreasing the half-life of osteopontin mRNA	[184]

Isoflavonoids	Genistein	Soy	HepG2, SMMC-7721, and Bel-7402 cells	Inhibit metastasis	Reversing the epithelial-mesenchymal transition, partly mediated by nuclear factor of activated T cell 1	[185]

Nonflavonoids	Resveratrol	Grapes, berries, red wine	HepG2 cells; xenograft model	Inhibit invasion and metastasis	Reducing MMP-9 via downregulation of NF-*κ*B signaling pathway; regulating HGF-c-Met signaling pathway	[186, 187]
	Theaflavins	Black tea	HepG2 and orthotopic model	Induce apoptosis; inhibit the growth and metastasis	Induce apoptosis by activating the caspase pathway; suppress the growth and metastasis through the blockage of STAT3 pathway	[188]
	(−)-Oleocanthal	Extravirgin olive oil	HCC cells; orthotopic HCC model	Inhibit growth and metastasis	Inhibiting STAT3 activation by decreasing JAK1 and JAK2 and enhancing SHP-1	[189]

*Anticarcinogenesis*						
Flavonols	Quercetin	Berries, apples, broccoli, beans, and tea	HepG2 cells	Anticarcinogenesis	Upregulation of p53 and BAX via downregulation of ROS, PKC, PI3K, and COX-2	[190]

Flavanols	EGCG	Tea		Anticarcinogenesis	Regulation of self-renewal Wnt/beta-catenin, Hh/Gli1 pathways and their associated genes cyclin D1, cMyc, and EGFR along with downregulation of E-cadherin	[146]

Flavonoid	Myricetin	Vegetables, fruits, nuts, berries, tea, and red wine	Animal with DEN-induced HCC	Inhibit the development of HCC	Inhibiting PAK1 via coordinate abrogation of MAPK/ERK and PI3K/AKT and their downstream signaling Wnt/*β*-catenin pathway	[191]

Nonflavonoids	Ellagic acid	Pomegranate, grapes, berries, walnuts, chocolate, wine, and green tea	Rats with N-nitrosodiethylamine-induced HCC	Anticarcinogenesis	Removing free radicals, preventing DNA fragmentation	[192]
	Curcumin	Ginger family		Anticarcinogenesis	Suppressing the protein expression of glypican-3, VEGF, and prothrombin	[86]

**Table 4:** Clinical trials of several polyphenols in liver diseases (refer to http://www.ClinicalTrials.gov website).

	Polyphenols	Status	Conditions	Purpose	Intervention	Phase	http://ClinicalTrials.gov identifier
NAFLD	Curcumin	Not yet recruiting	NAFLD patients with type 2 diabetes	Evaluate the effects of curcumin supplement on metabolic factors and hepatic fibrosis	1500 mg, 1 capsule/day for 12 weeks	2/3	NCT02908152
	Caffeine and chlorogenic acid	Not yet recruiting	NAFLD patients with type 2 diabetes	Investigate the effects of caffeine and chlorogenic acid supplements on inflammatory, metabolic factors, hepatic steatosis, and fibrosis	200 mg/d for 6 months	2/3	NCT02929901
	Resveratrol	Completed	Obese men with NAFLD	Evaluate potential metabolic effects of resveratrol	500 mg 3 times daily for six months	1	NCT01446276
	Resveratrol	Completed	Overweight adolescents with NAFLD and type 2 diabetes or metabolic syndrome	Demonstrate the safety and tolerability of resveratrol therapy	75 mg twice daily for a total daily dose of 150 mg for the duration of 30 days	2/3	NCT02216552
	Resveratrol	Completed	Obese patients with NAFLD/NASH	Evaluate the effects of resveratrol	500 mg 3 times daily for 6 months	1	NCT01464801
	Resveratrol	Completed	Patients with NAFLD/NASH	Evaluate effects of resveratrol supplement on biochemical factors and hepatic fibrosis	One resveratrol capsule per day for 12 weeks	2/3	NCT02030977

HCV	EGCG, silymarin	Completed	HCV patients	Determine the safety, metabolism, and antioxidant activity	Silymarin capsules, 700 mg, twice daily, 12 days; EGCG capsules, 196.5 mg, twice daily, 12 days	1	NCT01018615
	Quercetin	Completed	HCV patients	Evaluate safety and antiviral activity	2000 mg for 28 days	1	NCT01438320
	Naringenin	Completed	Persons infected with hepatitis C	To evaluate whether it can lower the amount of virus in the blood stream	1 gram of naringenin mixed with 16 grams of hydroxypropyl-*β*-cyclodextrin in 250 mL of water	1	NCT01091077

Cirrhosis and liver cancer	Green tea catechin extract	Not yet recruiting	Cirrhosis	Define how well it is in preventing liver cancer in patients with cirrhosis	Defined green tea catechin extract for 24 weeks	1	NCT03278925
	Resveratrol	Withdrawn	Liver cancer	Evaluate beneficial effect in the cellular function of normal liver cells and liver cancer cells	1 g daily for 10 days	1/2	NCT02261844
